# Comparison of the Impact of Insulin Degludec U100 and Insulin Glargine U300 on Glycemic Variability and Oxidative Stress in Insulin-Naive Patients With Type 2 Diabetes Mellitus: Pilot Study for a Randomized Trial

**DOI:** 10.2196/35655

**Published:** 2022-07-08

**Authors:** Pavle Vrebalov Cindro, Mladen Krnić, Darko Modun, Jonatan Vuković, Tina Tičinović Kurir, Goran Kardum, Doris Rušić, Ana Šešelja Perišin, Josipa Bukić

**Affiliations:** 1 Department of Gastroenterology University Hospital Split Split Croatia; 2 Department of Endocrinology University Hospital Split Split Croatia; 3 Department of Pathophysiology School of Medicine University of Split Split Croatia; 4 Department of Pharmacy School of Medicine University of Split Split Croatia; 5 Department of Psychology Faculty of Humanities and Social Sciences University of Split Split Croatia

**Keywords:** type two diabetes mellitus, type 2 diabetes mellitus, insulin degludec, insulin glargine U300, glucose variability, oxidative stress, insulin, diabetes, diabetic, glycemic variability, glycaemic variability, RCT, pilot, control trial, clinical trial

## Abstract

**Background:**

There is an ongoing discussion about possible differences between insulin degludec (IDeg-100) and glargine U300 (IGlar-300). There is little data and head-to-head comparison of IDeg-100 and IGlar-300 regarding their simultaneous impact on glycemic variability and oxidative stress in patients with type 2 diabetes mellitus (T2DM).

**Objective:**

In our randomized, open-label, crossover study, we compared the impact of IDeg-100 and IGlar-300 on glycemic variability and oxidative stress in insulin-naive patients with T2DM.

**Methods:**

We recruited a total of 25 adult patients with T2DM (7 females) whose diabetes was uncontrolled (HbA_1c_ ≥7.5%) on two or more oral glucose-lowering drugs; a total of 22 completed the study. Mean age was 57.3 (SD 6.99) years and duration of diabetes was 9.94 (SD 5.01) years. After the washout period, they were randomized alternately to first receive either IDeg-100 or IGlar-300 along with metformin. Each insulin was administered for 12 weeks and then switched. At the beginning and end of each phase, biochemical and oxidative stress parameters were analyzed. On 3 consecutive days prior to each control point, patients performed a 7-point self-monitoring of blood glucose profile. Oxidative stress was assessed by measuring thiol groups and hydroperoxides (determination of reactive oxygen metabolites test) in serum.

**Results:**

IGlar-300 reduced mean glucose by 0.02-0.13 mmol/L, and IDeg-100 reduced glucose by 0.10-0.16 mmol/L, with no significant difference. The reduction of the coefficient of glucose variation also did not show a statistically significant difference. IGlar-300 increased thiols by 0.08 µmol/L and IDeg-100 increased thiols by 0.15 µmol/L, with no significant difference (*P*=.07) between them. IGlar-300 reduced hydroperoxides by 0.040 CARR U and IDeg-100 increased hydroperoxides by 0.034 CARR U, but the difference was not significant (*P*=.12).

**Conclusions:**

The results of our study do not show a significant difference regarding glycemic variability between patients receiving either insulin IDeg-100 or IGlar-300, although IGlar-300 showed greater dispersion of data. No significant difference in oxidative stress was observed. In a larger study, doses of insulins should be higher to achieve significant impact on glycemic parameters and consequently on glycemic variability and oxidative stress.

**Trial Registration:**

ClinicalTrials.gov, NCT04692415; https://clinicaltrials.gov/ct2/show/NCT04692415

## Introduction

### Background

Global diabetes prevalence in 2019 is estimated to be 9.3% (463 million people), rising to 10.2% (578 million) by 2030 and 10.9% (700 million) by 2045 [1].

The main feature of diabetes mellitus of all types is dysglycemia, which consists of two main components: chronic sustained hyperglycemia and acute glycemic ﬂuctuations from peaks to nadirs. Although disputed by some authors [2], it is generally considered that both components contribute to diabetes complications through two main mechanisms—excessive protein glycation and activation of oxidative stress [3]—with glycemic variability being more specific in having an effect on oxidative stress than chronic sustained hyperglycemia [4], as both upward (postprandial glucose increments) and downward (interprandial glucose decrements) changes activate the oxidative stress pathway [5].

Glucose fluctuations gradually increase from normal glucose metabolism to impaired glucose regulation and diabetes mellitus. Intraday glucose variability occurs at the early stage of abnormal glucose tolerance. In addition to elevated intraday glucose fluctuations, newly diagnosed, drug-naive patients with type 2 diabetes mellitus (T2DM) also demonstrate increased postprandial glucose excursions, higher glucose levels overnight, and more interday fluctuations [6].

The main purpose of insulin therapy in diabetes mellitus is to control glucose—in other words, to combat dysglycemia. Long-acting basal insulin analogues (insulin glargine U100, insulin detemir) signiﬁcantly improved diabetes management, providing longer duration, ﬂatter proﬁles of action, lower risk of hypoglycemia, and less glycemic variability compared to NPH (Neutral Protamine Hagedorn) insulin [7,8].

The second generation of basal insulin analogues—insulin degludec 100 units/mL (IDeg-100) and insulin glargine 300 units/mL (IGlar-300)—have even smoother pharmacokinetic/pharmacodynamic proﬁles than insulin glargine U100, are longer acting, and further lower glycemic variability, at least in patients with T1DM [9,10].

Although several studies [11-13] have compared the impact of these two second-generation basal insulin analogues on glycemic variability in patients with type 1diabetes mellitus (T1DM), there is little data and head-to-head comparison of IDeg-100 and IGlar-300 regarding their simultaneous impact on glycemic variability and oxidative stress in patients with T2DM. In addition, the results from the T1DM studies are inconsistent [12,13].

### Aim of the Study

In this initial study, we compared the impact of IDeg-100 and IGlar-300 on glucose variability and oxidative stress (represented through its surrogate markers) in insulin-naive patients with T2DM. The main research question was whether there is any difference between the two insulins regarding these parameters. The results of this study should inform a larger study comparing these two insulins.

## Methods

### Ethics Approval and Consent to Participate

This randomized, open-label, crossover study was conducted in accordance with the Declaration of Helsinki and approved by the Ethics Committee of the University of Split School of Medicine (number 2181-198-03-04-17-0045). All subjects gave written consent prior to their participation in the study.

### Study Protocol and Population

**Figure 1:**
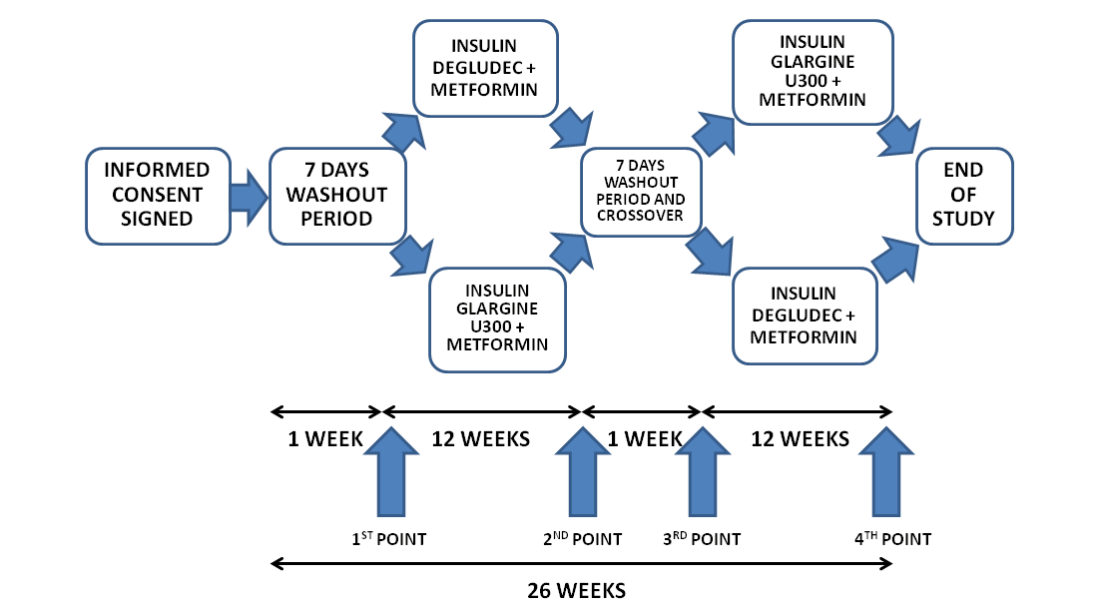
The study protocol.

Between December 2018 and May 2019, we recruited 25 outpatient insulin-naive patients with uncontrolled T2DM (glycated hemoglobin [HbA_1c_] ≥7.5% on two or more oral glucose-lowering drugs) and assigned them to either degludec insulin or glargine U300 insulin combined with metformin. All patients were recruited and treated at University Hospital Split, Croatia. All patients finished the study, but only 22 were analyzed (3 patients were excluded from data analysis—one patient did not perform his 7-point self-monitoring of blood glucose (SMBG) profile 3 days prior to control points, one patient decided to continue with his oral glucose-lowering agents only, and one patient left the study for personal reasons). Basal characteristics of the participants studied are shown in Table 1. The protocol of the study is shown in Figure 1. The study adheres to CONSORT (Consolidated Standards of Reporting Trials) guidelines (Multimedia Appendix 1 displays the CONSORT flow diagram). Patients and the public were not involved in the design, conduct, reporting, or dissemination plans of our research. The trial was retrospectively registered on ClinicalTrials.gov on December 31, 2020 (NCT04692415).

**Table 1:** Basal clinical characteristics of patients (N=25).

Parameter	Mean (SD)
Age (years)	56.23 (8.09)
Duration of diabetes (years)	8.90 (5.05)
Body weight (kg)	89.02 (13.82)
Body height (cm)	176.20 (10.03)
BMI (kg/m^2^)	28.92 (3.89)
Waist circumference (cm)	102.84 (8.56)
Glycated hemoglobin (HbA_1c_), %	9.66 (1.65)
Fasting glucose (mmol/L)	13.02 (4.47)
Serum creatinine (µmol/L)	68.24 (13.15)
Serum uric acid (µmol/L)	310.04 (60.75)
Total cholesterol (mmol/L)	5.28 (1.46)

Patients who were eligible for the study fulfilled all of the following inclusion criteria: history of T2DM for at least 1 year, aged between 45 and 65 years (women obligatory in postmenopause and with no hormonal replacement), uncontrolled glycemia on two or more oral antidiabetic drugs, no prior use of insulin, HbA_1c_ ≥7.5%, receiving statins (if not on statins, they were put on it), not on antiaggregant therapy (if on antiaggregants, they were temporarily excluded from therapy).

Exclusion criteria were the following: the use of glitazones or anticoagulant therapy, renal impairment with creatinine clearance <60 mL/s, presence of malignant disease, chronic liver disease, severe cardiovascular disease and history of cardiovascular incidents (stroke, myocardial infarction, peripheral amputation), and rheumatic and autoimmune diseases.

All participants were asked to avoid the consumption of vitamin supplements, coffee, wine, and Coca-Cola and similar beverages, especially in the days before each control point. Patients were also asked to avoid intensive physical activity up to two days before each control point. All subjects were told to report any side effects immediately, and were given the telephone numbers of the study conductors for this purpose. At each control point, participants were asked about possible side effects.

At baseline, all patients discontinued their previous therapy and were given metformin alone (2 g/day) for 7 days (washout period). After the 7-day washout period, they were randomized alternately by investigators (1:1 ratio) to first receive either IDeg-100 or IGlar-300 subcutaneously according to the order they were included in the study. In phase one, they received either IDeg-100 or IGlar-300 combined with metformin for 12 weeks. Phase one was followed by a second washout period in which patients received metformin alone again for 7 days. Finally, in phase two, which also lasted for 12 weeks, patients were switched from IDeg-100 to IGlar-300 and vice versa (and metformin was continued). The initial dose of both insulins was 0.2 IU/kg. We did not change the dose of insulin during the study period to avoid hypoglycemia, which could significantly influence the results [14-16].

At the beginning and end of each phase, blood samples were collected for the analysis of standard biochemical and oxidative stress parameters (control points 1-4). On 3 consecutive days prior to each control point (at the beginning and the end of each phase), patients completed the 7-point SMBG profile. All patients were already experienced with the use of SMBG [17].

### Glucose Measurement

To standardize results, all patients received a standard Bionime GM550 glucose meter. They were asked to regularly check their blood glucose 1-2 times per day during the entire study and, in the 3 consecutive days prior to each control point, to perform the 7-point SMBG profile. The 7-point blood glucose profile consisted of seven measurements: (1) before breakfast, (2) 2 hours after breakfast, (3) before lunch, (4) 2 hours after lunch, (5) before dinner, (6) 2 hours after dinner, and (7) before sleeping. Glucose variability was determined by calculating mean glycemia, SD, and coefficient of variation (CV) for each control point [17,18].

### Standard Laboratory Measurement

Serum uric acid concentrations, serum creatinine, total cholesterol, serum bilirubin values, and other basic biochemical laboratory values were determined by Olympus AU 600 Chemistry Analyzer (Olympus Michima Co Ltd) and enzymatic laboratory kit.

### Oxidative Stress Measurement

Thiol groups were assayed according to the Ellman assay [19], modified by Hu [20]. In detail, 100 µL of plasma was diluted with 2 mL of Tris-EDTA buffer (0.1 mol/L Tris,1 mmol/L EDTA, pH 8.2), and mixed with 100 µL of 10 mM DTNB (5,5′-dithiobis(2-nitrobenzoic acid)), previously prepared in methanol. To subtract the absorbance of plasma and DTNB at 412 nm, two parallel blank samples were assembled. The first one (“blank sample”) was prepared by mixing 2.1 mL of Tris-EDTA buffer with 100 μL of plasma and the second one (“blank reagent”) was prepared by mixing 2.1 mL of Tris-EDTA buffer with 100 μL of DTNB. All measurements were performed in triplicate and blanks were run for each sample. Readings were taken spectrophotometrically (Lambda 25; Perkin Elmer) at 412 nm after 15 minutes of reaction at 25 °C. Results were compared with a standard curve prepared daily with different concentrations of glutathione and expressed as µmol/L of glutathione.

The determination of reactive oxygen metabolites (d-ROM) assay measures the concentration of total hydroperoxides in serum or heparin plasma. The method was first described by Alberti et al in 1999 [21] and modified by Verde et al [22], and this modified assay was used to determine d-ROM values in plasma in this study. Each sample was prepared by mixing 2 mL of 0.1 M sodium acetate buffer (pH 4.8) and 20 µL of 0.1 M DMPD (N,N-diethyl-p-phenylenediamine) with 10 µL of plasma. After preparation, tubes with samples were vortexed for 15 seconds and incubated in a thermomixer (Thermomixer comfort, Eppendorf) at 37 °C and 1000 revolutions per minute for 75 minutes. All measurements were performed in triplicate and blanks were run for each sample. Readings were taken spectrophotometrically (Lambda 25; Perkin Elmer) at 505 nm after incubation. Results were compared with a standard curve prepared daily with different concentrations of H_2_O_2_. The results are expressed in CARR U (Carratelli units), where 1 CARR U corresponds to 0.08 mg/100 mL H_2_O_2_.

### Statistical Analysis

The number of subjects to include in the protocol was selected according to previously published literature [4,23]. Statistical analyses were performed using Statistica 6.0 (StatSoft Inc). Two-way ANOVA for repeated measures was used to evaluate changes in plasma glucose levels, CV, plasma thiols, and hydroperoxides due to IDeg-100 and IGlar-300.

## Results

Out of 25 randomized patients, 3 patients were excluded from data analysis—one patient did not perform his 7-point SMBG profile 3 days prior to control points, one patient decided to continue with his oral glucose-lowering agents only, and one patient left the study for personal reasons. No adverse reactions or unintended effects were noticed.

A total of 22 patients (7 females) successfully completed the trial, and their mean basal values were as follows: age, 57.3 (SD 6.99) years; duration of diabetes, 9.94 (SD 5.01) years; body weight, 88.25 (SD 13.57) kg; body height, 174.95 (SD 9.67) cm; BMI, 29.10 (SD 3.80) kg/m^2^; waist circumference, 102.73 (SD 8.02) cm; HbA_1c_, 9.60% (SD 1.68%); fasting glucose, 13.20 (SD 4.48) mmol/L; serum creatinine, 66.0 (SD 2.09) µmol/L; serum uric acid, 305.32 (SD 62.60) µmol/L; total cholesterol, 5.05 (SD 1.12) mmol/L.

**Figure 2:**
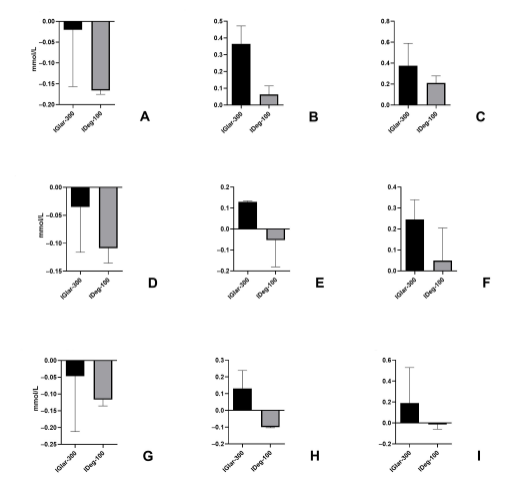
Mean glucose change, SD, and CV on the first, second, and third day. CV: coefficient of variation; IDeg-100: insulin degludec; IGlar-300: insulin glargine U300. A) Mean glucose change on the first day. B) SD of glucose change on the first day. C) CV of glucose change on the first day. D) Mean glucose change on the second day. E) SD of glucose change on the second day. F) CV of glucose change on the second day. G) Mean glucose change on the third day. H) SD of glucose change on the third day. I) CV of glucose change on the third day.

On the first of 3 consecutive days of 7-point SMBG, performed at the end of the observed period, IGlar-300 and IDeg-100 reduced mean glucose values by 0.02 and 0.16 mmol/L, respectively, which was statistically insignificant (*P*=.06; 95% CI 0.003 to 0.28); there was also no significant difference between the two insulins (*P*=.17; 95% CI –0.10 to 0.46) (Figure 2A).

The SD of glucose excursions was 0.36 for IGlar-300 and 0.06 for IDeg-100, which was not significant (*P*=.20; 95% CI –0.27 to 0.87); in addition, there was no statistically significant difference between the two insulins (*P*=.07; 95% CI –1.07 to 1.22) (Figure 2B)

The CV on the first day was 0.37 (37%) for IGlar-300 and 0.21 for IDeg-100, which was statistically insignificant (*P*=.22; 95% CI –0.30 to 0.63). When compared, CV for these two insulins was not significantly different (*P*=.20; 95% CI –0.73 to 1.15) (Figure 2C).

On the second of the 3 days of 7-point SMBG, performed at the end of the observed period, IGlar-300 and IDeg-100 reduced mean glucose by 0.03 and 0.10 mmol/L, respectively, which was statistically insignificant (*P*=.08; 95% CI –0.09 to 0.24); there was also no significant difference between the two insulins (*P*=.07; 95% CI –0.41 to 0.26) (Figure 2D).

The SD of glucose excursions on the second day was 0.12 for IGlar-300 and –0.05 for IDeg-100, which was insignificant (*P*=.19; 95% CI –0.23 to 0.59); when we compared the SD of the two insulins, there was no significant difference (*P*=.17; 95% CI –1.00 to 0.65) (Figure 2E).

The CV for the second day was 0.24 (24%) for IGlar-300 and 0.04 for IDeg-100 and that was statistically insignificant (*P*=.20; 95% CI –0.24 to 0.63). When compared, CV for the two insulins was not significantly different (*P*=.08; 95% CI –0.96 to 0.78) (Figure 2F).

On the third (last) day of the SMBG, the insulins reduced mean glucose levels by 0.04 (IGlar-300) and 0.11 mmol/L (IDeg-100), which was statistically insignificant (*P*=.08; 95% CI –0.10 to 0.24), again without a significant difference between the two insulins (*P*=.20; 95% CI –0.55 to 0.14) (Figure 2G).

The SD of glucose concentrations on the third day was 0.13 for IGlar-300 and –0.09 for IDeg-100, which was insignificant (*P*=.18; 95% CI –0.15 to 0.61), and comparison of the SD of the two insulins revealed no statistical difference (*P*=.14; 95% CI –0.62 to 0.91) (Figure 2H).

The CV on the third day was 0.19 for IGlar-300 and –0.01 for IDeg-100, which was statistically insignificant (*P*=.16; 95% CI –0.14 to 0.55). When compared, CV for these two insulins was not statistically different (*P*=.54; 95% CI –1.23 to 0.14) (Figure 2I).

**Figure 3:**
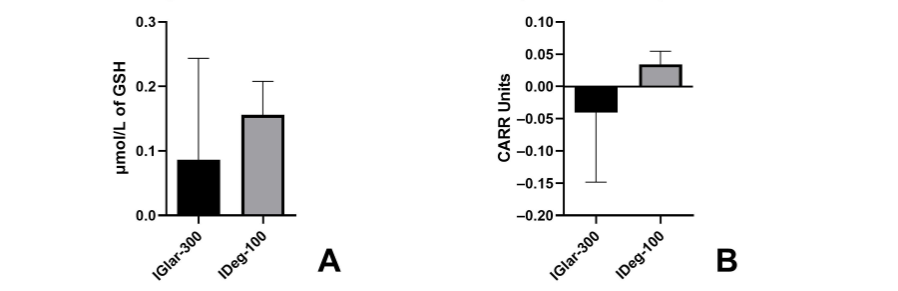
Changes in plasma thiols and hydroperoxides. GSH: glutathione; IDeg-100: insulin degludec; IGlar-300: insulin glargine U300. A) Changes in plasma thiols. B) Changes in plasma hydroperoxides.

IGlar-300 increased thiol levels by 0.08 µmol/L and IDeg-100 increased thiol levels by 0.15 µmol/L (*P*=.07; 95% CI –0.21 to 0.08). No significant difference was found between the two insulins regarding the increase of thiols (*P*=.14; 95% CI –0.15 to 0.44) (Figure 3A).

Although IGlar-300 decreased hydroperoxides by 0.04 CARR U, and IDeg-100 increased hydroperoxides by 0.034 CARR U (*P*=.06; 95% CI –0.19 to 0.05), this impact was not statistically significant, and there was no significant difference between the two insulins (*P*=.12; 95% CI –0.13 to 0.37) (Figure 3B).

## Discussion

### Principal Findings

Since the new generation of insulin analogues, degludec and glargine U300, appeared on the market, there has been an ongoing discussion about the possible advantages of one insulin over the other. The majority of comparisons related to the incidence of hypoglycemia [7,19,21-23], as well as the absorption stability and profile flatness, as possible causes of differences in hypoglycemia tendency were compared [7,13,24].

Absorption stability and profile flatness, if different, should lead to a difference in glycemic variability between the two insulins as variability in insulin absorption represents an important source of glucose variability in these subjects. Variability can relate to the insulin preparation, the injection technique, and the individual [25].

Glycemic variability and the incidence of hypoglycemia are the elements that “upgraded” the diabetological paradigm from the “diabetic triad” (HbA_1c_, fasting glycemia, and postprandial glycemia) to the “diabetic pentad” (HbA_1c_, fasting glycemia, postprandial glycemia, hypoglycemia, and glycemic variability) or even “hexad,” if quality of life is included [26].

The final consequences of increased glycemic variability and long-lasting hyperglycemia, as mentioned in the Introduction section, are diabetic complications, both micro- and macrovascular. As glycemic variability contributes to an increase in oxidative stress (one of two main mechanisms leading to the development of diabetic complications) to a greater extent, the need for the comparison of glycemic variability measures and oxidative stress markers in patients exposed to IDeg-100 and IGlar-300 emerges.

Two large, recently published studies (BRIGHT and DELIVER D+), although different in design, found “more similarities than differences” using IDeg-100 and IGlar-300. On the other hand, another large study (CONFIRM) attributed some advantages to IDeg-100. However, these studies, with the exception of the BRIGHT study, focused primarily on hypoglycemia rate and HbA_1c_ outcomes [27-29]. In this study, we wanted to associate glycemic variability with its ultimate consequence—oxidative stress.

Oxidative stress causes the development of diabetic complications through 5 main molecular mechanisms: the polyol pathway, the hexosamine pathway, increased formation of advanced glycation end products, increased expression of the receptors for advanced glycation end products and their activating ligands, and activation of protein kinase C isoforms. In addition, oxidative stress negatively influences the antiatherosclerotic endothelial enzymes: endothelial nitric oxide synthase and prostacyclin synthase. The intracellular reactive oxygen species increase by these mechanisms, then lead to defective angiogenesis in response to ischemia, and activate proinflammatory and epigenetic mechanisms after the normalization of glycemia (“hyperglycemic memory”) [30].

We used a basal-supported oral therapy variant in this study to emphasize the impact of the insulins studied. However, the results of this initial study did not show statistically significant differences both in glycemic variability and in the expression of oxidative stress in our patients. The primary reason for this was that the decrease in glycemic parameters was too small and, consequently, the impact on glycemic variability and oxidative stress was too weak. The too-small decrease in glycemic parameters was a consequence of using a low dose of insulin. Namely, we administered both insulins at a dose of 0.2 IU per kg of body weight, and we did not titrate the dose for two reasons: to avoid hypoglycemia, which could significantly influence the oxidative stress and glycemic variability results, and to eschew the difference in dosing of two insulins, which could also affect the results. Many studies have shown that hypoglycemia can worsen oxidative stress through, among other mechanisms, a decrease in nitric oxide and a “reperfusion-like” effect [14-16]. If an examinee has experienced hypoglycemia, he or she should be excluded from the study. Nevertheless, in future studies, the dose of insulins administered must be higher. The question is if “treat-to-target” is the best therapy approach, as treating to target inevitably lowers blood sugar toward hypoglycemia. We think that an initial dose of 0.4 IU/kg with very careful titration of the dose will achieve a more desirable glucose level, but still far from the hypoglycemic zone. A smaller-scale titration study to optimize the dose of insulin and the optimal time for oxidative marker readout would be a good in-between step.

Some studies showed a lower incidence of hypoglycemia with IDeg-100 versus IGlar-300, and that also could contribute to the lower levels of variability and oxidative stress observed with degludec [27], although other studies showed no difference between IDeg-100 and IGlar-300 in that regard [28,29]. Previous research has also suggested a somewhat greater potency of IDeg-100, thus titration to target would probably lead to differences in the final doses used and, consequently, make the comparison more difficult [24,28].

A longer exposure period (longer than 12 weeks) would allow for a more expressed impact of each insulin, presumed positive, although some studies showed a negative effect of chronic insulin therapy on oxidative stress [31]. The longer exposure would possibly explain the difference in the simultaneous increase in thiols and hydroperoxides produced by IDeg-100. The increase in thiol group concentrations represents protein oxidative stress reduction and d-ROM gives insight into the acute changes of lipid peroxide oxidation. This should be considered in the context of the negative effect of chronic insulin treatment on oxidative stress, as mentioned above.

We assessed within-day glycemic variability through changes in average glucose levels, SD of glycemic excursions, and the CV, derived from the SD. The 7-point SMBG profile represents the standard method of glucose monitoring, and we used it for the assessment of glycemic variability [17,32]. Diagnostic CGMS (continuous glucose monitoring systems) and the Libre Flash monitoring system would be more precise tools for glycemic variability measurement but, unfortunately, at the time, due to financial reasons, they could not be employed in this initial study. CGMS or the Flash monitoring system would allow the detection of a greater number of daily peaks and nadirs and give a better insight into glycemic variability [33]. Using CGMS, it would be possible to use the mean amplitude of glucose excursions (MAGE) index as an assessment tool for glycemic variability as well. Hence, in future studies, we highly recommend the use of CGMS.

In this study, we recruited insulin-naive patients who experienced the failure of oral glucose-lowering therapy and needed insulin introduction. We excluded those who were previously on pioglitazone therapy because of the prolonged action of this drug (which was impossible to remove during the 7-day washout period) and its possible influence on the results. Moreover, we standardized the concomitant therapy by introducing the same dose of atorvastatin in all patients and by temporarily removing salicylic acid from the therapy.

### Conclusion

The results of this study do not show a statistically significant difference in glycemic variability between IDeg-100 and IGlar-300. An insufficient dose of insulin was the main reason for the lack of impact on glycemic parameters and, consecutively, on glycemic variability. Probably due to the absence of a difference in glycemic variability, no difference in the oxidative stress level was noticed. A full-scale study should use larger doses of insulins (at least 0.4 IU/kg), and an optimized and adjusted “treat-to-target” algorithm. CGMS should be used instead of the 7-point SMBG profile. The MAGE index derived from the CGMS should be used for the assessment of glycemic variability. Another small titration study could be performed for optimization of the insulin dose and calculation of the sample size for the main study.

## Appendix

Multimedia Appendix 1 CONSORT flow diagram.

Multimedia Appendix 2 CONSORT 2010 checklist.

## Abbreviations

CGMS: continuous glucose monitoring system
CV: coefficient of variation
d-ROM: determination of reactive oxygen metabolites
HbA_1c_: glycated hemoglobin
IDeg-100: insulin degludec 100 IU/mL
IGlar-300: insulin glargine 300 IU/mL
MAGE: mean amplitude of glucose excursions
NPH: Neutral Protamine Hagedorn
SMBG: self-monitoring of blood glucose
T1DM: type 1 diabetes mellitus
T2DM: type 2 diabetes mellitus

## Data Availability Statement

The data sets used and/or analyzed in this study are available from the corresponding author on reasonable request.
