# Supplementary figures and images for: Comparison of the Impact of Insulin Degludec U100 and Insulin Glargine U300 on Glycemic Variability and Oxidative Stress in Insulin-Naive Patients With Type 2 Diabetes Mellitus: Pilot Study for a Randomized Trial
Source: JMIR Form Res. 2022 Jul 8;6(7):e35655. doi: 10.2196/35655 (PMC9308081; doi:10.2196/35655)

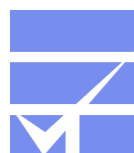

## CONSORT 2010 Flow Diagram

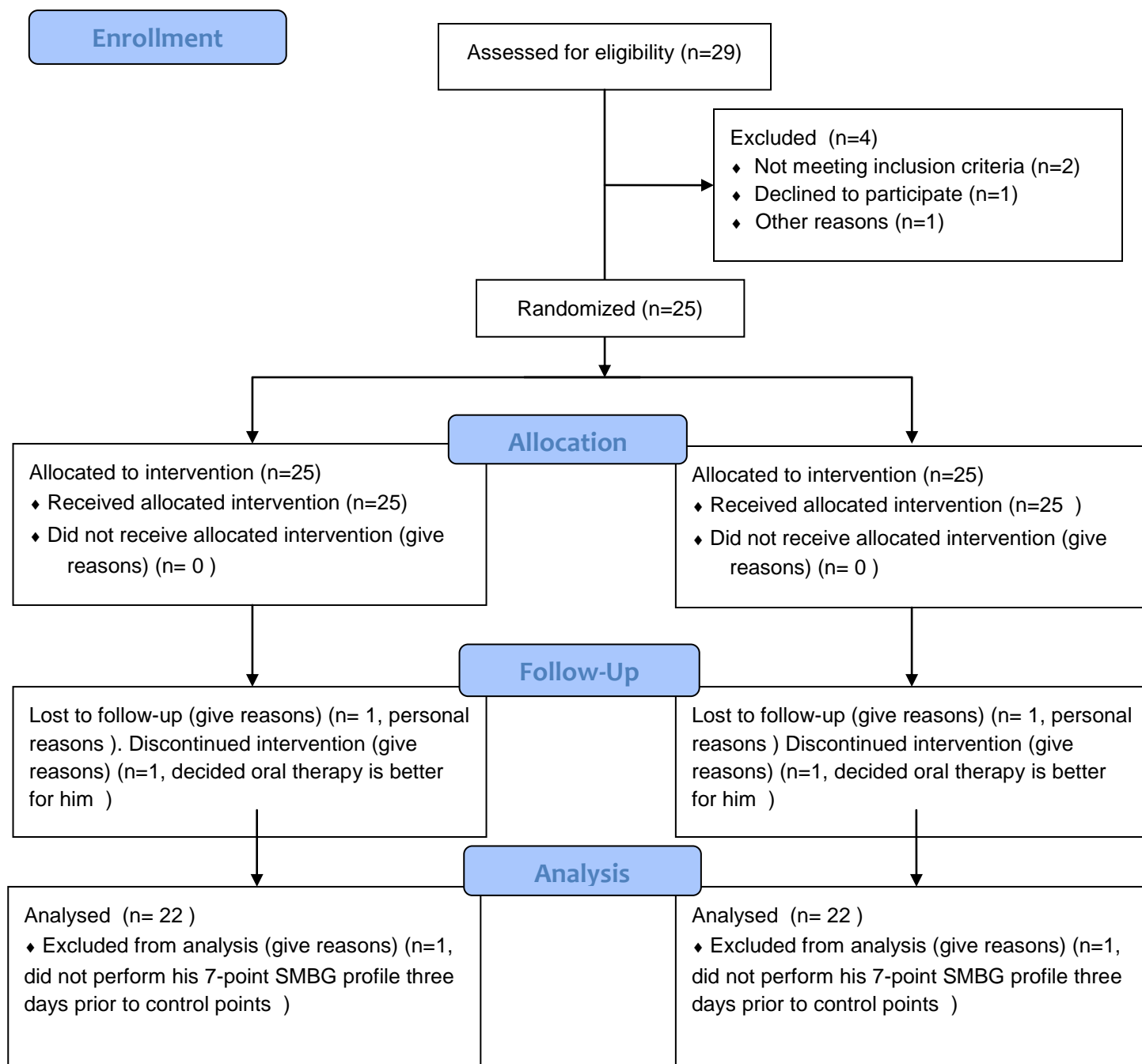

Supplement: Multimedia Appendix 1 [file formative_v6i7e35655_app1.pdf]
